# Associations between *RET* tagSNPs and their haplotypes and susceptibility, clinical severity, and thyroid function in patients with differentiated thyroid cancer

**DOI:** 10.1371/journal.pone.0187968

**Published:** 2017-11-13

**Authors:** Caiyun He, Jiangjun Ma, Yongle Jiang, Xuan Su, Xiao Zhang, Weichao Chen, Zulu Ye, Tiancheng Deng, Wenze Deng, Ankui Yang

**Affiliations:** 1 Department of Molecular Diagnostics, Sun Yat-Sen University Cancer Center, State Key Laboratory of Oncology in South China, Collaborative Innovation Center for Cancer Medicine, Guangzhou, China; 2 Sun Yat-Sen University, Guangzhou, China; 3 Department of Head and Neck, Sun Yat-sen University Cancer Center, State Key Laboratory of Oncology in South China, Collaborative Innovation Center for Cancer Medicine, Guangzhou, China; 4 Guangzhou University of Chinese Medicine, Guangzhou, China; Duke Cancer Institute, UNITED STATES

## Abstract

**Background:**

It is unclear whether common genetic variants of the *RET* proto-oncogene contribute to disease susceptibility, clinical severity, and thyroid function in differentiated thyroid cancer (DTC).

**Methods:**

A total of 300 DTC patients and 252 healthy controls were enrolled in this study. Seven RET tagging single nucleotide polymorphisms were genotyped using the KASPar platform.

**Results:**

Subgroup analysis showed that concomitant thyroid benign diseases were less likely to occur in DTC subjects with the rs1799939 AG or AG plus AA genotypes (odds ratio (OR) = 1.93 and 1.88, P = 0.009 and 0.011, respectively). A rare haplotype, CGGATAA, was associated statistically with a reduced risk of DTC (OR = 0.18, P = 0.001). Concerning the aggressive features of DTC, higher level of N stage was more likely to occur in subjects carrying the wild-type genotypes at rs1800860 site (for dominant model: OR = 0.48, P = 0.008). Another rare haplotype, CAAGCGT, conferred increased risk for the occurrence of distant metastasis (OR = 7.57, P = 0.009). Notably, higher thyroid stimulating hormone levels and lower parathyroid hormone levels were found in patients with rs2075912, rs2565200, and rs2742240 heterozygotes and rare homozygotes; similar results were observed between PTH levels and rs1800858.

**Conclusion:**

This study provided useful information on *RET* variants that should be subjected to further study.

## Introduction

Thyroid cancer, which is the most common malignancy in the endocrine system, is highly curable[1]. However, the increased incidence of differentiated thyroid carcinoma (DTC) is a serious problem[2]. Recent research has focused on the pathogenesis of this malignancy, with genetic factors being suggested to play an crucial role in the carcinogenesis of DTC[3]. Increasing efforts are being devoted to identifying susceptibility loci for this disease and its clinical phenotype. As the most common variants in the population, single nucleotide polymorphisms (SNPs) have the potential to be diagnostic, monitoring, and prognostic biomarkers for DTC.

The *RET* (rearranged during transfection) proto-oncogene has attracted much interest in DTC because aberrant activation of *RET* by somatic rearrangements or point mutations is a unique genetic event in DTC, which also plays a significant role in thyroid carcinogenesis[4, 5]. The protein encoded by *RET* functions as a multicomponent complex in which RET binds to a ligand belonging to the glial cell line–derived neurotrophic factor family[6]. In our previous work, we confirmed the association of *RET* rearrangements with DTC susceptibility and clinical phenotype in the Chinese population[7, 8]. To date, however, a systematic study of association between thecommon SNP of RET and susceptibility to DTC and its clinical phenotype has not been performed.

The levels of serum thyroid stimulating hormone (TSH), free triiodothyronine (fT3) and thyroxine (fT4), thyroglobulin (TG), and anti-thyroid peroxidase antibody (ATPO) are routine testing in the patients with thyroid cancer. These factors determine the balance of thyroid hormone homeostasis. Whether the common SNP in RET oncogene affects the thyroid function in DTC patients remains unclear and requires further study.

The *RET* gene is located at 10q11.2 and comprises 20 exons and 21 introns. To date, hundreds of SNPs in this gene have been revealed. However, information connecting *RET* SNPs with susceptibility to DTC is very limited. To assess comprehensively the role of *RET* genetic polymorphism, we adopted a candidate gene association study strategy with seven selected potentially functional tagging SNPs (tagSNPs) in *RET*[9]. We examined their individual and combined effects on the risk of DTC and their relationship with clinical severity and thyroid function.

## Material and methods

### Study subjects

This protocol was approved by the Ethics Committee of Sun Yat-sen University Cancer Center, Guangdong, China. Written informed consent was obtained from all patients at their first visit. For the genetic association study, a total of 552 subjects, comprising 300 DTC cases and 252 healthy control subjects, were included. The blood collection took place when the patients first visited this center. All the included subjects were recruited from Sun Yat-sen University Cancer Center in Guangzhou of Guangdong Province, China, between July 2011 and June 2016. We excluded two DTC patients from further analysis because of a past history of breast cancer or lung cancer. The healthy subjects comprised individuals with normal manifestation of the thyroid gland and thyroid function under ultrasound and serum examination. Healthy subjects who had a history of nodular goiter, Hashimoto's thyroiditis, or other benign thyroid diseases, and who had a history of other malignancies, were excluded.

### Data collection

Medical records and surgical pathology reports were reviewed by Caiyun He, Yongle Jiang and Xuan Su. Information of demographic parameters, pathological characteristics of the tumors, and thyroid function parameters were collected. Slides were reviewed independently by two pathologists to confirm the diagnosis of DTC and concomitant thyroid benign diseases, such as nodular goiter and Hashimoto's thyroiditis. Information on the primary tumor size, stage grouping, extrathyroid extension, and metastasis were assessed based on the National Comprehensive Cancer Network (NCCN Guidelines, Version 1, 2016) on thyroid cancer recommendations (https://www.nccn.org/). The clinicopathological features of T, N and M stages and extrathyroid extension are well-known parameters indicating the clinical severity of thyroid cancer. The measurement and distribution of the clinicopathological features of thyroid cancer patients were showed in S1 Table.

### TagSNP selection

HapMap genotype data of the Chinese Han Beijing (CHB) population (Release 27, Phase I +II +III, http://www.HapMap.org) were extracted within extended gene regions of *RET* in 2016, which encompassed 2 kb of upstream and downstream flanking sequence. The CHB *RET* gene has 33 common SNPs. As described in our previous studies, tagSNPs were selected by using the Haploview software[10, 11]. Briefly, tagSNPs were chosen initially based on pairwise linkage disequilibrium information to maximally represent (r2 > 0.8) common SNPs (minor allele frequency (MAF) > 0.05) by Haploview 4.2. We then used bioinformatics search to prioritize the tagSNPs for genotyping, based on their predicted functional effects (http://snpinfo.niehs.nih.gov/). For example, rs1799939 was predicted to be located at a splicing site, suggesting a potential effect on this gene's splicing regulation.

### DNA isolation and genotyping of RET tagSNPs

For each subject, 3 mL of fasting blood was collected for DNA isolation. Genomic DNA was isolated from peripheral blood lymphocytes by the routine phenol–chloroform method, as previously described[10, 11]. Each DNA sample was diluted to a working concentration of 50 ng/μL for genotyping. Assay design and SNP genotyping were performed by Gene Company Limited (Shanghai, China) using the KASPar platform according to the manufacturer’s instructions[12]. All samples were randomized on 384-well plates and blinded for disease status. Twenty randomly selected samples were genotyped repeatedly, and the results were 100% concordant.

### Statistical analysis

The Hardy-Weinberg Equilibrium (HWE) of the genotype distribution of each SNP was detected by a chi-square test in the control group. The odds ratio (OR) and corresponding 95% confidence interval (CI) were calculated to measure the strength of the association between the genotype and the risk of DTC. The genetic effect of a single tagSNP on disease risk was detected by Logistic regression analysis controlling for sex and age. For the haplotype analysis of the seven RET tagSNPs (rs17028, rs1799939, rs1800858, rs1800860, rs2075912, rs2565200, and rs2742240), we set the other haplotypes pooled together as a reference, and assessed the genetic effect of each haplotype with a frequency of at least more than 0.03 in the healthy controls.

For the association analysis between the genotype and clinicopathological features, we only performed association analysis in DTC patients since the control subjects have normal manifestation of thyroid. Logistic regression analysis adjusting for sex and age was employed. For the analysis of thyroid function, the difference of fT3, fT4, TSH, Anti-TG, TG, PTH and calcitonin levels between two groups were compared by the Mann–Whitney U test because these quantitative variables did not follow a normal distribution. The corresponding variables are shown as the median (with 25–75% quartiles).

All these analyses were performed using SPSS 17.0 software (SPSS, Chicago, IL, USA), except for the haplotype analysis, which was performed using the online software SHEsis[13] (http://analysis.bio-x.cn/myAnalysis.php). All P values were two sided, and a P value < 0.05 was considered statistically significant. The authenticity of this article has been validated by uploading the key raw data onto the Research Data Deposit public platform (www.researchdata.org.cn), with the approval RDD number as RDDB2017000172.

## Results

### Functional prediction of tagSNPs

Seven tagSNPs in *RET* with potential functions were included in this study and their predicted functional effects are summarized in Table 1. Among the seven tagSNPs, rs1799939, rs1800858, and rs1800860 are located within exon regions and were predicted to affect exonic splicing enhancer (ESE) or exonic splicing silencer (ESS) binding site activity, or even abolish a protein domain. The other four tagSNPs, rs17028, rs2075912, rs2565200, and rs2742240 are located in 3’ untranslated region (UTR) and were predicted to be located within microRNA (miRNA) binding sites. The distribution of the genotype frequencies of the seven tagSNPs in healthy controls obeyed the Hardy-Weinberg equilibrium test (Table 1, all P > 0.05). Therefore, these seven tagSNPs were included for the association analysis.

**Table 1:** Functional prediction of seven tagSNPs of the RET gene.

SNP	Position	Allele	TFBS	Splicing (ESE or ESS)	miRNA	nsSNP	HWE
rs17028	3' UTR	C/T	—	—	Y	—	0.123
rs1799939	exon	A/G	—	Y	—	Y	0.864
rs1800858	exon	A/G	—	Y	—	—	0.945
rs1800860	exon	A/G	—	Y	—	—	0.258
rs2075912	3'UTR	C/T	—	—	Y	—	0.391
rs2565200	3'UTR	T/C	—	—	Y	—	0.320
rs2742240	3'UTR	A/T	—	—	Y	—	0.319

Abbreviation: ESE, splicing Enhancers; ESS, exonic Splicing Silencers; HWE, Hardy–Weinberg equilibrium; nsSNP, nonsynonymous single nucleotide polymorphism; miRNA, microRNA; RET, rearranged during transfection proto-oncogene; tagSNP, tagging single nucleotide polymorphism; TFBS, transcription factor binding site; UTR, untranslated region; Y, yes.

### Association between RET tagSNPs and susceptibility, clinicopathological features, and thyroid function of DTC

When we considered the influence of the *RET* tagSNPs on susceptibility to DTC, none of the seven tagSNPs showed an association with risk of DTC (Table 2, all P > 0.05). In the subgroup analysis, rs1799939 AG or AG plus AA genotypes showed an association with increased risk for the DTC cases without concomitant thyroid benign diseases (OR = 1.93 and 1.88, respectively, see S2 Table). No association was found between RET SNPs and the risk of DTC cases with concomitant thyroid benign diseases (see S3 Table).

**Table 2:** Association between RET tagSNP and susceptibility to thyroid cancer.

TagSNP	Control (%)	Cancer (%)	OR (95%CI)	*P for overall*	*P*
**rs17028**					0.186^a^
CC	139 (57.0%)	174 (59.2%)	1 (ref)		
TC	96 (39.3%)	101 (34.4%)	0.80 (0.56–1.16)		0.239
TT	9 (3.7%)	19 (6.5%)	1.65 (0.72–3.79)		0.240
Dominant model			0.89 (0.63–1.25)		0.493
Recessive model			1.79 (0.79–4.06)		0.161
**rs1799939**					0.703 ^a^
GG	194 (78.2%)	220 (75.1%)	1 (ref)		
AG	51 (20.6%)	69 (23.5%	1.19 (0.79–1.80)		0.407
AA	3 (1.2%)	4 (1.4%)	1.08 (0.24–4.92)		0.920
Dominant model			1.19 (0.79–1.78)		0.403
Recessive model			1.10 (0.24–5.01)		0.898
**rs1800858**					0.880 ^a^
GG	77 (30.8%)	87 (29.8%)	1 (ref)		
GA	123 (49.2%)	143 (49.0%)	1.08 (0.73–1.60)		0.715
AA	50 (20.0%)	62 (21.2%)	1.13 (0.69–1.84)		0.628
Dominant model			1.07 (0.74–1.55)		0.709
Recessive model			1.10 (0.72–1.68)		0.655
**rs1800860**					0.463 ^a^
GG	143 (57.9%)	187 (63.2%)	1 (ref)		
GA	94 (38.1%)	99 (33.4%)	0.80 (0.56–1.14)		0.220
AA	10 (4.0%)	10 (3.4%)	0.85 (0.34–2.11)		0.721
Dominant model			0.80 (0.57–1.14)		0.218
Recessive model			0.93 (0.38–2.29)		0.873
**rs2075912**					0.371 ^a^
CC	63 (25.7%)	86 (29.1%)	1 (ref)		
CT	129 (52.7%)	139 (47.0%)	0.79 (0.53–1.19)		0.259
TT	53 (21.6%)	71 (24.0%)	1.00 (0.62–1.63)		0.986
Dominant model			0.85 (0.58–1.25)		0.410
Recessive model			1.18 (0.79–1.77)		0.425
**rs2565200**					0.313 ^a^
GG	64 (25.9%)	86 (29.3%)	1 (ref)		
GA	131 (53.0%)	138 (46.9%)	0.78 (0.52–1.18)		0.238
AA	52 (21.1%)	70 (23.8%)	1.03 (0.63–1.67)		0.909
Dominant model			0.85 (0.58–1.25)		0.414
Recessive model			1.22 (0.81–1.83)		0.349
**rs2742240**					0.361 ^a^
TT	65 (26.1%)	85 (28.9%)	1 (ref)		
TA	132 (53.0%)	139 (47.3%)	0.80 (0.54–1.20)		0.284
AA	52 (20.9%)	70 (23.8%)	1.05 (0.64–1.70)		0.855
Dominant model			0.88 (0.60–1.28)		0.496
Recessive model			1.22 (0.81–1.84)		0.340

^a^, the overall p-value for 2-df comparison of the three genotype groups for each SNP. Logistic regression analysis adjusting for sex and age was implemented. Abbreviation: ref, reference; tagSNP, tagging single nucleotide polymorphism; OR, odds ratio.

We further analyzed the relationship between *RET* tagSNPs and the clinicopathological features of DTC (Table 3). When compared with the CC or CC plus TC genotypes, TT variants at rs17028 was boarderline associated with a reduced risk of developing a higher T stage exceeding T1 (for TT *vs*. CC: OR = 0.26, 95% CI: 0.08–0.85, P = 0.026; for TT *vs*. CC and TC: OR = 0.25, 95% CI: 0.08–0.78, P = 0.017), and seemed to associated with a reduced risk for the occurrence of extrathyroid extension (for TT *vs*. CC: OR = 0.34, 95% CI: 0.11–1.00, P = 0.050; for TT *vs*. CC and TC: OR = 0.34, 95% CI: 0.12–0.97, P = 0.044). The rs1800860 GA variant had a 0.45-fold reduced risk for the occurrence of lymph node metastasis compared with the common GG genotype (P = 0.005); a similar effect was observed for GA plus AA variants (OR = 0.48, 95% CI: 0.28–0.83, P = 0.008).

**Table 3:** Association between RET tagSNP and clinicopathological features of thyroid cancer.

TagSNP	T stage		*P*	N stage		*P*	M stage		*P*	Extrathyroid extension		*P*
	No. of T1/T2–4	OR (95%CI)		No. of N0/N1	OR (95%CI)		No. of M0/M1	OR (95%CI)		No. of No/Yes	OR (95%CI)	
**rs17028**												
CC	69/105	1 (ref)		56/118	1 (ref)		164/10	1 (ref)		89/84	1 (ref)	
TC	37/64	0.75 (0.45–1.26)	0.278	35/66	0.88 (0.51–1.52)	0.640	94/7	1.18 (0.43–3.25)	0.752	49/51	1.09 (0.66–1.80)	0.747
TT	14/5	0.26 (0.08–0.85)	0.026	4/15	0.90 (0.56–6.45)	0.304	19/0	/	/	14/5	0.34 (0.11–1.00)	0.050
Dominant model		1.06 (0.66–1.71)	0.817		0.97 (0.57–1.63)	0.896		0.98 (0.36–2.69)	0.974		0.92 (0.57–1.48)	0.724
Recessive model		0.25 (0.08–0.78)	0.017		1.95 (0.59–6.47)	0.277		/	/		0.34 (0.12–0.97)	0.044
**rs1799939**												
GG	92/128	1 (ref)		69/151	1 (ref)		210/10	1 (ref)		112/106	1 (ref)	
AG	30/39	0.98 (0.56–1.72)	0.941	26/43	0.60 (0.33–1.11)	0.102	63/6	2.10 (0.71–6.15)	0.178	40/29	0.76 (0.44–1.34)	0.349
AA	0/4	3.95 (0.38–40.82)	0.249	0/4	/	/	4/0	/	/	0/4	/	/
Dominant model		1.07 (0.62–1.85)	0.818		0.68 (0.37–1.22)	0.196		1.99 (0.68–5.83)	0.209		0.89 (0.52–1.54)	0.680
Recessive model		4.75 (0.45–50.08)	0.194		/	/		/	/		/	/
**rs1800858**												
GG	38/49	1 (ref)		27/60	1 (ref)		84/3	1 (ref)		41/45	1 (ref)	
GA	56/87	1.14 (0.65–1.97)	0.654	49/94	0.87 (0.47–1.62)	0.664	134/9	1.78 (0.46–5.93)	0.407	75/67	0.89 (0.51–1.55)	0.687
AA	27/35	0.70 (0.35–1.41)	0.317	16/46	1.52 (0.68–3.41)	0.308	58/4	2.12 (0.43–10.42)	0.357	35/27	0.71 (0.35–1.42)	0.333
Dominant model		0.98 (0.58–1.66)	0.953		1.06 (0.59–1.89)	0.852		1.92 (0.52–7.07)	0.329		0.82 (0.49–1.39)	0.464
Recessive model		0.61 (0.34–1.11)	0.103		1.68 (0.86–3.28)	0.133		1.24 (0.38–4.07)	0.719		0.78 (0.44–1.40)	0.404
**rs1800860**												
GG	70/117	1 (ref)		50/137	1 (ref)		176/11	1 (ref)		94/92	1 (ref)	
GA	49/50	0.78 (0.47–1.28)	0.322	42/57	**0.45 (0.26–0.78)**	**0.005**	93/6	1.03 (0.37–2.89)	0.957	56/42	0.72 (0.44–1.20)	0.210
AA	2/8	1.80 (0.44–7.45)	0.415	3/7	0.71 (0.16–3.13)	0.654	10/0	/	/	3/7	1.93 (0.47–7.97)	0.361
Dominant model		0.84 (0.52–1.37)	0.490		**0.48 (0.28–0.83)**	**0.008**		0.91 (0.33–2.56)	0.863		0.80 (0.49–1.30)	0.370
Recessive model		2.19 (0.54–8.81)	0.272		1.26 (0.31–5.23)	0.748		/	/		2.25 (0.56–9.09)	0.254
**rs2075912**												
CC	36/50	1 (ref)		31/55	1 (ref)		83/3	1 (ref)		43/43	1 (ref)	
CT	56/83	0.12 (0.64–1.95)	0.691	41/98	1.23 (0.67–2.26)	0.509	130/9	1.95 (0.50–7.560	0.335	72/65	1.00 (0.58–1.74)	0.999
TT	29/42	0.92 (0.48–1.77)	0.795	23/48	1.28 (0.62–2.63)	0.505	66/5	2.13 (0.48–9.45)	0.321	38/33	0.92 (0.48–1.77)	0.806
Dominant model		1.04 (0.62–1.75)	0.872		1.27 (0.72–2.23)	0.407		2.03 (0.56–7.33)	0.281		0.97 (0.58–1.62)	0.892
Recessive model		0.83 (0.47–1.44)	0.500		1.12 (0.61–2.07)	0.711		1.44 (0.48–4.34)	0.515		0.95 (0.55–1.65)	0.859
**rs2565200**												
GG	35/51	1 (ref)		30/56	1 (ref)		83/3	1 (ref)		42/44	1 (ref)	
GA	56/82	1.04 (0.60–1.82)	0.877	39/99	1.24 (0.67–2.29)	0.491	130/8	1.71 (0.43–6.74)	0.444	72/64	0.93 (0.54–1.62)	0.806
AA	28/42	0.89 (0.46–1.72)	0.724	23/47	1.19 (0.58–2.45)	0.644	65/5	2.20 (0.49–9.82)	0.301	37/33	0.90 (0.47–1.73)	0.750
Dominant model		0.99 (0.59–1.66)	0.965		1.25 (0.71–2.20)	0.441		1.89 (0.52–6.86)	0.336		0.92 (0.55–1.54)	0.740
Recessive model		0.84 (0.48–1.47)	0.537		1.05 (0.57–1.94)	0.883		1.62 (0.53–4.97)	0.396		0.97 (0.56–1.69)	0.913
**rs2742240**												
TT	35/50	1 (ref)		31/54	1 (ref)		82/3	1 (ref)		42/43	1 (ref)	
TA	57/82	1.07 (0.62–1.87)	0.809	40/99	1.27 (0.69–2.35)	0.438	131/8	1.67 (0.42–6.61)	0.466	73/64	0.95 (0.54–1.66)	0.855
AA	28/42	0.90 (0.46–1.73)	0.744	23/47	1.25 (0.61–2.56)	0.551	65/5	2.20 (0.49–9.77)	0.301	37/33	0.91 (0.47–1.75)	0.769
Dominant model		1.02 (0.61–1.71)	0.945		2.30 (0.74–2.28)	0.365		1.85 (0.51–6.75)	0.350		0.94 (0.56–1.58)	0.938
Recessive model		0.86 (0.49–1.50)	0.586		1.09 (0.59–2.01)	0.783		1.63 (0.53–4.98)	0.393		0.99 (0.57–1.72)	0.972

Logistic regression analysis adjusting for sex and age was implemented. Associations that reached statistical significance were highlighted in bold.

We also explored whether *RET* variants affect pre-operative thyroid function in DTC patients (Table 4). Variants in the rs2075912, rs2565200 and rs2742240 loci were associated statistically with increased thyroid stimulating hormone (TSH) levels but decreased parathyroid hormone (PTH) levels. The PTH levels in DTC cases carrying GA genotype at rs1800858 site were also statistically lower than those in the wild-type carriers. In addition, subjects carrying the rare rs17028 TT homozygote genotype showed a borderline association with a higher PTH levels compared with the common CC homozygotes.

**Table 4:** Association between RET tagSNP and pre-operative thyroid function.

TagSNP	freq	fT3		fT4		TSH		ATPO		Anti-TG		TG		PTH		Calcitonin	
		Median	*P*^*a*^	Median	*P*^*a*^	Median	*P*^*a*^	Median	*P*^*a*^	Median	*P*^*a*^	Median	*P*^*a*^	Median	*P*^*a*^	Median	*P*^*a*^
Median (25%,75%) value for the total DTC patients		1.7(4.3,5.1)		16.8(15.1,18.9)		2.0(1.3,2.9)		11.1(7.1,21.3)		21.2(7.4,50.2)		21.2(7.4,50.2)		36.6(27.8,46.0)		24.4(22.0,27.6)	
**rs17028**																	
CC	168	4.66		16.67		2.15		11.30		21.01		20.50		35.66		24.75	
TC	97	4.61	0.884	17.52	0.359	1.99	0.205	10.82	0.669	19.36	0.729	19.83	0.959	34.88	0.796	23.83	0.124
TT	19	4.66	0.689	16.79	0.364	1.71	0.105	8.90	0.338	19.69	0.916	26.17	0.151	39.52	0.048	23.77	0.528
Dominant model			0.795		0.608		0.098		0.489		0.734		0.670		0.673		0.118
Recessive model			0.703		0.221		0.177		0.351		0.988		0.142		0.043		0.765
**rs1799939**																	
GG	212	4.65		16.66		2.04		10.62		21.40		21.72		35.56		24.39	
AG	68	4.70	0.876	16.94	0.693	1.98	0.521	12.96	0.578	18.35	0.222	20.14	0.891	37.18	0.078	24.90	0.653
AA	4	4.54	0.602	17.75	0.809	2.59	0.269	10.38	0.916	160.42	0.117	55.71	0.436	36.46	0.556	23.06	0.891
Dominant model			0.965		0.666		0.696		0.607		0.392		0.978		0.069		0.685
Recessive model			0.571		0.828		0.224		0.842		0.086		0.427		0.619		0.912
**rs1800858**																	
GG	85	4.66		16.78		1.92		12.39		18.73		25.51		39.19		24.04	
GA	140	4.66	0.913	16.94	0.200	2.15	0.230	10.98	0.699	20.10	0.657	18.97	0.101	33.72	**0.003**	24.33	0.806
AA	58	4.66	0.583	16.49	0.548	2.27	0.129	10.84	0.845	20.13	0.997	25.09	0.681	37.28	0.192	25.17	0.562
Dominant model			0.897		0.233		0.137		0.830		0.737		0.166		**0.006**		0.684
Recessive model			0.545		0.982		0.358		0.730		0.819		0.646		0.864		0.614
**rs1800860**																	
GG	180	4.65		16.52		2.12		10.66		21.01		23.82		35.66		24.52	
GA	96	4.69	0.968	17.28	0.121	2.00	0.581	11.59	0.738	19.78	0.859	18.24	0.323	35.94	0.614	24.12	0.893
AA	10	4.73	0.708	15.48	0.318	2.18	0.601	12.71	0.904	17.08	0.309	24.94	0.938	40.96	0.224	26.10	0.948
Dominant model			0.895		0.230		0.518		0.778		0.930		0.366		0.437		0.913
Recessive model			0.739		0.238		0.716		0.811		0.288		0.827		0.232		0.892
**rs2075912**			0.572		0.554		**0.014**		0.763		0.933		0.747		**0.017**		0.559
CC	84	4.67		16.82		1.83		11.32		19.06		22.56		38.54		23.83	
CT	136	4.64	0.772	16.76	0.277	2.12	**0.013**	11.20	0.474	19.60	0.874	19.96	0.434	34.50	**0.005**	24.61	0.377
TT	66	4.70	0.428	16.76	0.491	2.30	**0.008**	10.75	0.636	21.43	0.792	23.91	0.649	37.32	0.163	24.96	0.315
Dominant model			0.904		0.285		**0.004**		0.476		0.996		0.452		**0.009**		0.293
Recessive model			0.306		0.843		0.162		0.938		0.729		0.884		0.885		0.552
**rs2565200**																	
GG	85	4.66		16.81		1.83		11.29		19.19		23.40		38.57		23.77	
GA	135	4.65	0.997	16.78	0.210	2.12	**0.015**	11.26	0.367	19.52	0.778	19.83	0.630	34.68	**0.004**	24.58	0.379
AA	65	4.70	0.359	16.65	0.436	2.31	**0.010**	10.74	0.589	21.50	0.826	23.25	0.618	23.58	0.116	24.94	0.357
Dominant model			0.706		0.219		**0.005**		0.381		0.911		0.385		**0.007**		0.309
Recessive model			0.311		0.838		0.172		0.956		0.715		0.902		0.932		0.630
**rs2742240**																	
TT	83	4.68		16.81		1.84		11.29		19.19		23.40		38.57		23.77	
TA	136	4.64	0.853	16.76	0.263	2.12	**0.017**	11.20	0.368	19.60	0.914	19.76	0.306	34.50	**0.006**	24.51	0.443
AA	65	4.70	0.402	16.65	0.478	2.31	**0.012**	10.74	0.572	21.50	0.766	23.25	0.582	37.30	0.129	24.94	0.387
Dominant model			0.833		0.271		**0.006**		0.377		0.965		0.333		**0.010**		0.363
Recessive model			0.295		0.841		0.190		0.940		0.721		0.903		0.956		0.614

^a^, Analysis performed using the Mann-Whitney test. Associations that reached statistical significance were highlighted in bold. Abbreviation: ATPO, anti-thyroid peroxidase antibody; freq, frequency; fT3, free triiodothyronine; fT4, free thyroxine; tagSNP, tagging single nucleotide polymorphism; TG, thyroglobulin; TSH, thyroid stimulating hormone; PTH, Parathyroid hormone.

### Association between haplotype of RET and clinicopathological features and risk of DTC

For DTC susceptibility, a rare haplotype of CGGATAA of rs17028-rs1799939-rs1800858-rs1800860-rs2075912-rs2565200-rs2742240 showed a significant association with reduced risk of developing DTC, demonstrating an OR of 0.18 (95% CI: 0.06–0.54, P values for Pearson’s Chi-square test or Fisher’s exact test = 0.001, Table 5).

**Table 5:** Association between haplotypes of RET tagSNPs and susceptibility to thyroid cancer.

Haplotype	Case (%)	Control (%)	OR (95% CI)	Fisher's *P*	Pearson's *P*
C G A G T A A	234 (41.8%)	183 (40.4%)	1.12 (0.86–1.45)	0.405	0.405
T G G G C G T	110 (19.6%)	90 (19.8%)	1.02 (0.74–1.40)	0.905	0.905
C A G A C G T	55 (9.8%)	48 (10.6%)	0.95 (0.63–1.44)	0.810	0.810
C G G A C G T	33 (5.8%)	28 (6.2%)	0.95 (0.57–1.61)	0.859	0.859
C G G G C G T	48 (8.6%)	43 (9.5%)	0.92 (0.60–1.42)	0.706	0.706
**C G G A T A A**	**4 (0.7%)**	**18 (3.9%)**	**0.18 (0.06–0.54)**	**0.001**	**0.001**
C G G G T A A	22 (3.9%)	11 (2.3%)	1.81 (0.85–3.84)	0.118	0.118

Associations that reached statistical significance were highlighted in bold.

For the clinicopathological features of DTC, another rare haplotype CAAGCGT of rs17028-rs1799939-rs1800858-rs1800860-rs2075912-rs2565200-rs2742240 conferred increased risk for the occurrence of distant metastasis (OR = 7.57, 95% CI = 1.01–56.56, P values for Pearson’s Chi-square test or Fisher’s exact test = 0.009, Table 6). No associations were observed between haplotypes of the seven *RET* tagSNPs and T stage, N stage, extrathyroid extension, number of cancer lesions, and occurrence of concomitant thyroid benign diseases in DTC (all P > 0.05, data not shown).

**Table 6:** Association between haplotypes of RET tagSNPs and the occurrence of distant metastasis in thyroid cancer.

Haplotype	Case (%)	Control (%)	OR (95% CI)	Fisher's P	Pearson's P
**C A A G C G T**	**1 (3.6%)**	**3 (0.5%)**	**7.57 (1.01–56.56)**	**0.009**	**0.009**
C A G A C G T	5 (17.9%)	50 (9.4%)	1.92 (0.70–5.26)	0.200	0.200
C G A G T A A	14 (50.0%)	220 (41.6%)	1.23 (0.57–2.63)	0.597	0.597
C G G G C G T	1 (3.6%)	47 (8.9%)	0.35 (0.05–2.62)	0.284	0.284
C G G G T A A	1 (3.6%)	20 (3.8%)	0.88 (0.16–4.82)	0.788	0.788
T G G G C G T	6 (21.4%)	102 (19.4%)	1.03 (0.41–2.61)	0.947	0.947

Associations that reached statistical significance were highlighted in bold.

## Discussion

Genetic variants of the proto-oncogene *RET* have attracted much research attention in recent studies of cancer causation[4, 5]. Our evaluation of a subgroup of DTC patients without concomitant thyroid benign diseases showed that DTC was more likely to occur in subjects with the rs1799939 AG or AG plus AA genotypes compared with the common GG genotype. Concerning the aggressive features of DTC, higher level of N stage was more likely to exist in subjects carrying the common homozygotes of rs1800860. Of note, higher TSH levels and lower PTH levels were found in patients with rs2075912, rs2565200, and rs2742240 heterozygotes and rare homozygotes, and similar results were observed between PTH levels and rs1800858.

We observed that the carriers with rs1799939 variants were more susceptible to DTC without concomitant thyroid benign diseases. rs1799939 is one of the most frequently studied SNPs in *RET*, and leads to an amino acid change from glycine to serine. In this study, online informatics software also predicted that rs1799939 would affect the splicing of *RET* transcripts. In another Chinese study, Huang and Yang reported that the rs1799939 AA genotype conferred a 3.76-fold increased risk of thyroid cancer[14]. A positive association was also reported in Khan et al.’s study in an Indian population[15]. However, a negative association for this SNP was found in Santos et al.’s study in a Portuguese Population[16]. Besides an independent effect of a single SNP, we observed an association between a rare haplotype, CGGATAA, (incorporated by rs17028-rs1799939-rs1800858-rs1800860-rs2075912-rs2565200-rs2742240 SNPs) and reduced risk of DTC, compared with other haplotypes, including the A risky allele at the rs1799939 site, which excluded chance as an explanation for the observed associations. The mechanisms by which the *RET* polymorphisms may confer an increased susceptibility to DTC remain to be determined. A pathogenic role of this SNP in DTC should be further evaluated *in vitro*.

We also found relationships between two RET tagSNPs and the aggressive features of DTC. The homozygous genotype of rs17028 showed hints of association with a reduced risk of the occurrence of a higher T stage, as well as extrathyroid extension, and the heterozygous genotype of rs1800860 contributed to a reduced risk for the occurrence of lymph node metastasis. rs17028 and rs1800860 have been reported in patients with Hirschsprung disease[17]. In addition, rs1800860 was reported to be associated with the risk of differentiated thyroid cancer[18]. This is an initial study reporting the frequencies of rs17028 and rs1800860 and their relationship with the clinicopathological features in DTC. As for the molecular function, a study focusing on kidney size in newborns suggested that rs1800860 was identified within an exonic splicing enhancer[19]. As predicted in this study, the variant A in the mRNA reduced the affinity for spliceosome proteins, enhanced the likelihood of aberrant mRNA splicing, and diminished the level of the functional transcript in human cells. So far, there is no known function for rs17028. The variant T allele of rs17028, located in the *RET* 3’ UTR, was predicted to impair the binding affinity of miR-24. He et al.’s study suggested overexpression of miR-24 in DTC[20]. Therefore, we hypothesized that the weakened binding affinity of miR-24 might constrain tumor progression in DTC patients harboring the T allele. Whether this SNP affects *RET* function in DTC in this manner requires further study.

In contrast to the susceptibility and clinicopathological features, *RET* variants have rarely been evaluated in thyroid function. Among the clinical parameters of thyroid function, the most frequently affected factors are TSH and PTH, which are critical factors for monitoring patients after surgery. Our previous study found that *RET* fusion genes correlated with higher TSH levels after surgery. In this study, we found several SNPs that were related to pre-operative thyroid function, including rs2075912, rs2565200, rs2742240, and rs1800858. Strikingly, these four SNPs did not show an association with susceptibility and clinical phenotype of DTC, whilst the DTC risk and clinical phenotype associated rs1799939 and rs1800860 were not related with thyroid function. This phenomenon provoked speculation that *RET* variants exclusively affect the development of DTC and thyroid function. Among the four SNPs, rs2075912, rs2565200, and rs2742240, are located in the *RET* 3’ UTR and were predicted to affect the binding of certain miRNAs. This suggested a biological function of the *RET* variants in the regulation of miRNAs that affect the synthesis of thyroid factors. Although the SNPs were not related to the susceptibility and TNM staging in DTC, it remains unclear whether these *RET* SNPs could predispose patients the recurrence of DTC, which requires further study.

It is a pity that there are several limitations in our study. In this study, the statistical analyses were performed on seven RET SNPs. And our study population is relatively small, especially for the haplotype analysis for distant metastasis in thyroid cancer; thereby, impact of RET SNPs on disease risk and disease severity might be probably underestimated. View in this manner, we considered a more conservative significance level of 0.05 rather than 0.05/7, as recommended previously[21, 22]. However, we are aware that the statistical results of our analyses should be considered to be exploratory, and hence did not adjust for multiple testing.

In conclusion, DTC without concomitant thyroid benign diseases was more likely to occur in subjects with the rs1799939 AG variant compared with those carrying the common GG genotype. An association was found between the rare haplotype CGGATAA and reduced risk of DTC compared with other haplotypes incorporating the risky A allele at rs1799939 site, which excluded chance as an explanation for the observed associations. A higher level of N stage was more likely to exist in subjects carrying the wild-type genotype at rs1800860. Notably, higher TSH levels and lower PTH levels were found in patients with rs2075912, rs2565200, and rs2742240 heterozygotes and rare homozygotes, and similar results were observed between PTH levels and the GA genotype of rs1800858.

## Supporting information

S1 Table Measurement and distribution of the clinicopathological features of thyroid cancer patients. (DOCX)

S2 Table Association between RET tagSNP and susceptibility to thyroid cancer in DTC patients without concomitant thyroid benign diseases. (DOCX)

S3 Table Association between RET tagSNP and susceptibility to thyroid cancer in DTC patients with concomitant diseases. (DOCX)
