# Supplementary material for: Associations between RET tagSNPs and their haplotypes and susceptibility, clinical severity, and thyroid function in patients with differentiated thyroid cancer
Source: PLoS One. 2017 Nov 13;12(11):e0187968. doi: 10.1371/journal.pone.0187968 (PMC5683616; doi:10.1371/journal.pone.0187968)
Supplement: S1 Table — (DOCX) [file pone.0187968.s001.docx]

**S1 Table Measurement and distribution of the clinicopathological features of thyroid cancer patients**

| Parameters | Frequency(%) |
| --- | --- |
| **T stage** |  |
| 1(≤2cm) | 122(40.9%) |
| 2(2-4cm) | 38(12.8%) |
| 3(≥4cm) | 128(43.0%) |
| 4(advanced disease) | 10(3.4%) |
| **N stage** |  |
| 0(no regional lymph node metastasis) | 95(31.9%) |
| 1(regional lymph node metastasis) | 203(68.1% |
| **M stage** |  |
| 0(no distant metastasis) | 281(94.3%) |
| 1(distant metastasis) | 17(5.7%) |
| **Extrathyroid extension** |  |
| 0(no any minimal extension) | 160(53.7%) |
| 1(extension to sternothyroid muscle, perithyroid soft tissues, or extending beyond) | 138(46.3%) |
