# Supplementary material for: Associations between RET tagSNPs and their haplotypes and susceptibility, clinical severity, and thyroid function in patients with differentiated thyroid cancer
Source: PLoS One. 2017 Nov 13;12(11):e0187968. doi: 10.1371/journal.pone.0187968 (PMC5683616; doi:10.1371/journal.pone.0187968)
Supplement: S2 Table — (DOCX) [file pone.0187968.s002.docx]

**S2 table Association between RET tagSNP and susceptibility to thyroid cancer in DTC patients without concomitant thyroid benign diseases**

| TagSNP | Control(%) | Cancer(%) | OR(95%CI) | *P* |
| --- | --- | --- | --- | --- |
| **rs17028** |  |  |  |  |
| CC | 139(57.0%) | 74(58.3%) | 1(ref) |  |
| TC | 96(39.3%) | 46(36.2%) | 0.88(0.55–1.39) | 0.577 |
| TT | 9(3.7%) | 7(5.5%) | 1.22(0.43-3.52) | 0.71 |
| TT+TC vs. CC |  |  | 0.92(0.59-1.43) | 0.701 |
| TT vs. CC+TC |  |  | 1.36(0.49-3.81) | 0.554 |
| **rs1799939** |  |  |  |  |
| GG | 194(78.2%) | 84(66.7%) | 1(ref) |  |
| AG | 51(20.6%) | 41(32.5%) | **1.93(1.18-3.16)** | **0.009** |
| AA | 3(1.2%) | 1(0.8%) | 0.83(0.08-8.28) | 0.874 |
| AA+AG vs. GG |  |  | **1.88(1.16-3.06)** | **0.011** |
| AA vs. GG+AG |  |  | 0.76(0.78-7.52) | 0.815 |
| **rs1800858** |  |  |  |  |
| GG | 77(30.8%) | 41(32.8%) | 1(ref) |  |
| GA | 123(49.2%) | 62(49.6%) | 0.98(0.60-1.62) | 0.945 |
| AA | 50(20.0%) | 22(17.6%) | 0.85(0.45-1.62) | 0.626 |
| AA+GA vs. GG |  |  | 0.93(0.59-1.49) | 0.774 |
| AA vs. GG+GA |  |  | 0.84(0.48-1.48) | 0.547 |
| **rs1800860** |  |  |  |  |
| GG | 143(57.9%) | 79(62.7%) | 1(ref) |  |
| GA | 94(38.1%) | 43(34.1%) | 0.82(0.52-1.29) | 0.388 |
| AA | 10(4.0%) | 4(3.2%) | 0.92(0.27-3.11) | 0.891 |
| AA+GA vs. GG |  |  | 0.83(0.53-1.29) | 0.408 |
| AA vs. GG+GA |  |  | 1.02(0.31-3.40) | 0.974 |
| **rs2075912** |  |  |  |  |
| CC | 63(25.7%) | 38(30.2%) | 1(ref) |  |
| CT | 129(52.7%) | 61(48.4%) | 0.78(0.47-1.30) | 0.342 |
| TT | 53(21.6%) | 27(21.4%) | 0.87(0.47-1.64) | 0.672 |
| TT+CT vs. CC |  |  | 0.81(0.50-1.31) | 0.385 |
| TT vs. CC+CT |  |  | 1.00(0.59-1.71) | 0.999 |
| **rs2565200** |  |  |  |  |
| GG | 64(25.9%) | 38(30.6%) | 1(ref) |  |
| GA | 131(53.0%) | 60(48.0%) | 0.77(0.46-1.28) | 0.308 |
| AA | 52(21.1%) | 27(21.6%) | 0.91(0.49-1.71) | 0.776 |
| AA+GA vs. GG |  |  | 0.81(0.50-1.31) | 0.385 |
| AA vs. GG+GA |  |  | 1.05(0.61-1.79) | 0.859 |
| **rs2742240** |  |  |  |  |
| TT | 65(26.1%) | 38(30.2%) | 1(ref) |  |
| TA | 132(53.0%) | 61(48.4%) | 0.78(0.47-1.30) | 0.343 |
| AA | 52(20.9%) | 27(21.4%) | 0.90(0.48-1.69) | 0.745 |
| AA+TA vs. TT |  |  | 0.82(0.51-1.33) | 0.423 |
| AA vs. TT+TA |  |  | 1.04(0.61-1.77) | 0.899 |

Associations that reached statistical significance were highlighted in bold.
