# Supplementary material for: Associations between RET tagSNPs and their haplotypes and susceptibility, clinical severity, and thyroid function in patients with differentiated thyroid cancer
Source: PLoS One. 2017 Nov 13;12(11):e0187968. doi: 10.1371/journal.pone.0187968 (PMC5683616; doi:10.1371/journal.pone.0187968)
Supplement: S3 Table — (DOCX) [file pone.0187968.s003.docx]

**S3 table Association between RET tagSNP and susceptibility to thyroid cancer in DTC patients with concomitant diseases**

| TagSNP | Control(%) | Cancer(%) | OR(95%CI) | *P* |
| --- | --- | --- | --- | --- |
| **rs17028** |  |  |  |  |
| CC | 139(57.0%) | 100(59.9%) | 1(ref) |  |
| TC | 96(39.3%) | 55(32.9%) | 0.73(0.48–1.12) | 0.153 |
| TT | 9(3.7%) | 12(7.2%) | 1.89(0.76–4.69) | 0.170 |
| TT+TC vs. CC |  |  | 0.85(0.56–1.27) | 0.420 |
| TT vs. CC+TC |  |  | 2.11(0.86–5.15) | 0.102 |
| **rs1799939** |  |  |  |  |
| GG | 194(78.2%) | 136(81.4%) | 1(ref) |  |
| AG | 51(20.6%) | 28(16.8%) | 0.79(0.47–1.31) | 0.357 |
| AA | 3(1.2%) | 3(1.8%) | 1.31(0.26–6.62) | 0.746 |
| AA+AG vs. GG |  |  | 0.82(0.50–1.34) | 0.429 |
| AA vs. GG+AG |  |  | 1.43(0.28–7.19) | 0.665 |
| **rs1800858** |  |  |  |  |
| GG | 77(30.8%) | 46(27.5%) | 1(ref) |  |
| GA | 123(49.2%) | 81(48.5%) | 1.13(0.71–1.80) | 0.605 |
| AA | 50(20.0%) | 40(24.0%) | 1.36(0.78–2.37) | 0.281 |
| AA+GA vs. GG |  |  | 1.17(0.76–1.81) | 0.480 |
| AA vs. GG+GA |  |  | 1.29(0.80–2.07) | 0.296 |
| **rs1800860** |  |  |  |  |
| GG | 143(57.9%) | 108(63.5%) | 1(ref) |  |
| GA | 94(38.1%) | 56(32.9%) | 0.79(0.52–1.20) | 0.272 |
| AA | 10(4.0%) | 6(3.5%) | 0.82(0.29–2.36) | 0.714 |
| AA+GA vs. GG |  |  | 0.79(0.53–1.19) | 0.260 |
| AA vs. GG+GA |  |  | 0.89(0.32–2.52) | 0.828 |
| **rs2075912** |  |  |  |  |
| CC | 63(25.7%) | 48(28.1%) | 1(ref) |  |
| CT | 129(52.7%) | 78(45.9%) | 0.80(0.50–1.27) | 0.338 |
| TT | 53(21.6%) | 44(25.7%) | 1.10(0.63–1.91) | 0.739 |
| TT+CT vs. CC |  |  | 0.88(0.57–1.37) | 0.576 |
| TT vs. CC+CT |  |  | 1.31(0.82–2.07) | 0.260 |
| **rs2565200** |  |  |  |  |
| GG | 64(25.9%) | 48(28.4%) | 1(ref) |  |
| GA | 131(53.0%) | 78(46.2%) | 0.79(0.50–1.27) | 0.333 |
| AA | 52(21.1%) | 43(25.4%) | 1.10(0.63–1.93) | 0.729 |
| AA+GA vs. GG |  |  | 0.88(0.57–1.37) | 0.581 |
| AA vs. GG+GA |  |  | 1.32(0.83–2.11) | 0.243 |
| **rs2742240** |  |  |  |  |
| TT | 65(26.1%) | 47(28.0%) | 1(ref) |  |
| TA | 132(53.0%) | 78(46.4%) | 0.82(0.51–1.31) | 0.401 |
| AA | 52(20.9%) | 43(25.6%) | 1.14(0.65–1.99) | 0.650 |
| AA+TA vs. TT |  |  | 0.92(0.59–1.43) | 0.703 |
| AA vs. TT+TA |  |  | 1.34(0.84–2.13) | 0.220 |
